# A study on the effects of modified sprint interval trainingon physical fitness test scores and the quantitative and dose-response relationships among Chinese male university students

**DOI:** 10.3389/fphys.2025.1555019

**Published:** 2025-02-25

**Authors:** Guoyuan Huang, Yang Chen, ByungChan Lee, Yipeng Qiu, Aqiang Mao, Maolong Liang, Maojie Liu

**Affiliations:** ^1^ School of Physical Education, Sichuan Agricultural University, Yaan, China; ^2^ Ya’an Key Laboratory of Sports Human Science and National Fitness Promotion, Yaan, China; ^3^ Chungwoon University Department of Physical education, Hongseong, South Chungcheong, Republic of Korea; ^4^ College of Information Engineering, Sichuan Agricultural University, Ya’an, Sichuan, China

**Keywords:** sprint interval training, exercise intervention, college students, physical fitness, physical fitness test

## Abstract

**Introduction:**

This study primarily investigates the impact of a 6-week Sprint Interval Training (SIT) intervention on the physical fitness test results of male university students, as well as the dose-response relationship in adjusting the experimental protocol.

**Methods:**

A total of 26 male university students (aged 20 ± 2 years; height 174 ± 7 cm; weight 70 ± 14 kg; mean ± SD) with no systematic training in the past 3 months, no physiological diseases, and healthy physical condition voluntarily participated in the experiment. The SIT protocol was designed based on a classic Wingate sprint protocol (4-6 x 30 s sprints with 4 m of recovery), and adjustments were made based on the participants' actual adaptation. The final intervention consisted of 6 weeks of training, three times per week, with 2-3 repetitions of 30-s Wingate sprints (Cd = 0.075, resistance on the ergometer = weight/kg x Cd) and 4–5 m of recovery.

**Results:**

The results showed significant improvements in key anaerobic capacity indicators after the 6-week intervention: Average Power (AP) increased from 77.4 ± 10.1 to 132.6 ± 21.1 (p < 0.01, Adjusted p < 0.03 ment, with a maximum effect size of 3.344), Peak Power (PP) increased from 102.9 ± 14.5 to 189.5 ± 28.8 (p < 0.01, Adjusted p < 0.02, maximum effect 3.790), and Time to Peak Power (TTP) decreased from 12.3 ± 3.3 to 9.5 ± 2.6 (p < 0.01), confirming that the intervention enhanced the participants' anaerobic capacity. Additionally, The results of the physical fitness test showed significant improvements: standing long jump (SLJ) increased from 2.31 ± 0.15 m to 2.45 ± 0.18 m (significance level p < 0.01), 50 m sprint time decreased from 7.32 ± 0.42 s to 6.98 ± 0.38 s (significance level p < 0.01), and 1,000 m from 235.6 ± 18.4 s to 220.3 ± 16.8 s (significance level p < 0.01). Other metrics such as Body mass, BMI, Vital capacity, and Pull-ups also showed minor increases. Interestingly, Sit forward in a sitting position scores showed a noticeable improvement (from 12.9 ± 6.8 to 15.8 ± 6.2, p = 0.091).

**Discussion:**

Furthermore, The adjustment of the training programme has achieved good results, as evidenced by the fact that participants have achieved a training completion rate of over 95%, maintained a moderate subjective fatigue rating (RPE score of 13-15), and no one has withdrawn from the training due to discomfort.

**Conclusion:**

The modified SIT protocol proves to be an efficient and practical training method for improving college students' physical fitness.

## 1 Introduction

Currently, college students generally face suboptimal health conditions, and physical fitness tests can reveal students’ underlying health issues, encouraging them to engage in physical exercise and promoting the development of sports culture in universities,At the same time, physical fitness test results serve as an important indicator to measure the health level of college students (Wang, 2019; Zhao et al., 2024). Recent research has demonstrated that physical fitness test scores are significantly influenced by students’ sports participation habits, highlighting the importance of understanding and improving students’ exercise patterns (Yu, 2024). While physical activity interventions have shown promising effects on students’ mental health and wellbeing, recent studies indicate that only a small proportion of universities provide structured physical activity programs for mental health promotion (Malagodi et al., 2024). The decline in physical fitness among college students is caused by a combination of multiple internal and external factors. The physical health status of college students faces various challenges, and both domestic and international students show a decline in physical fitness. The reasons for this include academic pressure, life stress, sedentary behavior, lack of exercise, poor diet, and irregular lifestyle, all of which contribute to the deterioration of college students’ physical health (Pan et al., 2022; Zhang and Xu, 2023). Recent data from Chinese Double First-Class universities revealed that the COVID-19 pandemic has significantly worsened this situation, with the total “fail” percentage in physical fitness tests increasing from 9.19% in 2019 to 12.94% in 2021, particularly affecting students’ endurance and strength qualities (Jiang et al., 2024). In response, scientific exercise training should be adopted to increase the frequency of exercise, establish a health-first mindset, and promote the concept of lifelong physical exercise. Only through continuous exploration of scientific and practical exercise methods can the physical health of college students be effectively improved (Liu et al., 2023).

One of the main factors hindering exercise among college students is the lack of time, with recent research demonstrating that increasing academic workload and screen-based learning activities have significantly contributed to students’ sedentary behavior and reduced physical activity participation (Wei et al., 2023). Sprint Interval Training (SIT) is considered an innovative and efficient training method that can induce rapid changes in exercise capacity and skeletal muscle energy metabolism. It promotes neural adaptation in a short period through comprehensive neurophysiological mechanisms, including enhanced motor unit recruitment efficiency and muscle contraction coordination. These adaptations typically occur within specific time windows, with initial neural responses appearing within hours of training, followed by more sustained adaptations in neuromuscular function and pain tolerance mechanisms (Greenhouse-Tucknott et al., 2022) and increases the levels of muscle creatine (CR) and creatine kinase (CK), optimizing muscle oxidative capacity, muscle glycogen content, and muscle buffering ability (Burgomaster et al., 2008; Gibala et al., 2006; Buchheit et al., 2010). Additionally, SIT can enhance athletic performance and improve metabolic health (Whyte et al., 2010; Billat, 2001; Denham et al., 2015). Through structured periodic SIT training, anaerobic gains can be strengthened, and cardiovascular function can be rapidly improved, enhancing heart and lung health (Denham et al., 2015; Farley et al., 2016). SIT is a variation of interval training, typically consisting of 4-6 sets of 30-s all-out Wingate sprints with 4 m of rest in between (Bostad et al., 2021; Burgomaster et al., 2005). The duration of each exercise session is short, with actual exercise time only around 2–3 m per set, and the total time per session is around 18–27 m. This time-efficient training method may address the issue of lack of time to some extent. Recent meta-analysis has shown that even low-volume high-intensity interval training (≤5 min high-intensity exercise within a ≤15 min session) can significantly improve cardiorespiratory fitness and body composition while requiring only 14%–47% of the time commitment compared to traditional moderate-intensity continuous training (MICT) (Yin et al., 2023). Studies have shown that SIT can achieve similar health benefits in only one-fifth of the time required for traditional MICT (Gillen et al., 2016). Recent systematic reviews and meta-analyses have demonstrated that even short-term SIT interventions can achieve moderate improvements across various physical performance outcomes, particularly in anaerobic capacity (Hall et al., 2023). A randomized controlled trial (RCT) by Burgomaster et al. also confirmed that SIT is effective compared to traditional endurance training (Burgomaster et al., 2008).

Moreover, as a subtype of High-Intensity Interval Training (HIIT), SIT shares many benefits with HIIT. Both SIT and HIIT can stimulate a series of adaptive responses in skeletal muscles and have positive effects on controlling blood sugar, improving cardiovascular health, and enhancing both anaerobic and aerobic fitness (Gibala and McGee, 2008; Babraj et al., 2009; Buchheit and Laursen, 2013; Buchheit et al., 2008). However, HIIT is not suitable for all populations, as it is typically used by elite athletes to improve performance and is generally not recommended for the general population. Traditionally, aerobic exercise for the general population has been limited to moderate-intensity endurance activities, with recommended durations ranging from 30–60 m daily or 20–60 m of vigorous exercise (Bull et al., 2020). In contrast, SIT’s short and efficient nature meets the exercise time needs of the general population. Therefore, this study investigates whether a six-week SIT intervention can influence various indicators of male college students’ physical fitness test scores and explores the dose-response relationship by adjusting the training protocol.

## 2 Methods

### 2.1 Subjects

A total of 26 male university students (age: 20 ± 2 years; height: 174 ± 7 cm; weight: 70 ± 14 kg; mean ± SD) participated in this study. The sample size was determined based on a statistical power analysis for the primary outcome measure (anaerobic power), with a significance level of α = 0.05, a power (1-β) of 0.80, and an effect size of 0.5 (medium effect size based on previous SIT studies), which resulted in a minimum required sample size of 27 for paired t-test analysis. Taking into account a possible dropout rate of around 10%, 26 participants were ultimately recruited. Participants were recruited through school bulletin boards and physical education classes using a convenience sampling method. All selected participants had not undergone systematic training in the past three months26. It can also significantly improve maximum oxygen uptake (4.2%–13.4%) (Gist et al., 2014) and aerobic capacity (∼8%) (Sloth et al., 2013). were free from any physiological diseases, and had healthy physical conditions, The selection criteria for the subjects were as follows: 1. Inclusion criteria: (1) Healthy male college students aged 18-22; (2) Body mass index (BMI) in the range of 18.5–24.9 kg/m^2^; (4) Physical examination report showing normal indicators, including: resting heart rate: 60–100 beats/min; blood pressure: systolic pressure <140 mmHg, diastolic pressure <90 mmHg. 2. Exclusion criteria: (1) a history of cardiovascular, respiratory or metabolic disease; (2) injury to the musculoskeletal system within the past year; (3) taking medication that affects exercise capacity; (4) regular smoking or drinking habits; (5) participation in other sports experiments or training programmes. All participants were required to complete pre-experiment assessment and screening, including a physical examination, the Physical Activity Readiness Questionnaire (PAR-Q), and signing the informed consent form for the experiment. voluntarily participated in the experiment after signing an informed consent form that covered the precautions and potential issues during the study. Participants were instructed to reduce their own physical activity during the study period to minimize the risk of injury and ensure the accuracy of the intervention. Additionally, participants were required to refrain from alcohol consumption, overeating, or fasting for at least 48 h before the experiment. They were also asked to have a reasonable meal at least 3 h prior to the test to avoid conducting the test on an empty stomach. As a study conducted in China, this research was carried out as part of normal educational and training activities in accordance with Article 32 of the ‘Ethical Review Measures for Life Sciences and Medical Research Involving Humans’ jointly issued by the National Health Commission of China, the Ministry of Education, the Ministry of Science and Technology, and the National Administration of Traditional Chinese Medicine in February 2023.

### 2.2 Study design

This study employed a pre-post experimental design without randomization or control group. This design choice was made considering: (1) the preliminary exploratory nature of the study focusing on SIT’s impact on college students’ physical fitness; (2) limited research resources and time; (3) recruitment challenges. We acknowledge this as a limitation that may affect result interpretation and will address it in future research with a randomized controlled design. To ensure the reliability of the research results, this study follows a standardised method for quantifying external load (Dhahbi et al., 2024a)and uses a proven test protocol to evaluate training results (Dhahbi et al., 2017; Čular et al., 2021; Padulo et al., 2019)The research team is aware that this limitation in the design may affect the interpretation of the results, so more caution will be taken when analysing the results.

The choice of 6 weeks as the intervention period in this study was based on the evidence from existing studies. Studies have shown that a 6-week SIT intervention can produce significant physiological adaptations, including metabolic adaptations and improvements in exercise performance (Sarkar et al., 2021; MacInnis et al., 2017; MacInnis and Gibala, 2017). Although this duration may not fully reflect the long-term adaptation effect, as a preliminary exploratory study, the 6-week intervention period can provide important reference for understanding the impact of SIT on the physical fitness of college students.

Although numerous studies have used the classic SIT protocol (4-6 x 30 s Wingate sprints) for experiments, it has not been proven to be the optimal SIT protocol, and the number of repetitions and duration remain arbitrary (Vollaard and Metcalfe, 2017). Therefore, this study adopted the classic protocol as a reference, using 4-6 repetitions of 30-s Wingate sprints with 4 m of recovery (Gibala et al., 2014). Based on the “National Physical Fitness and Health Standard for Students,” physical fitness tests for male university students are divided into three categories: body composition (BMI), physical function (vital capacity), and physical fitness (strength and speed). With anaerobic power (including Average Power and Peak Power) designated as the primary outcome measure, The intervention included SIT training over a set period, with physical fitness tests conducted before and after the intervention for comparative analysis.

This study modified the traditional SIT programme as follows, based on three main considerations: 1. Exercise tolerance: Pre-experiments found that college students have poor tolerance for the traditional 4-6 sets programme, and they become significantly fatigued after completing 2-3 sets. Studies have shown that even if it is reduced to 2-3 sets, significant metabolic effects can still be achieved (Vollaard and Metcalfe, 2017), and blood oxygen saturation can be increased by about 14% (Allemeier et al., 1994). 2. Training safety: The reason for using the ‘2 + 3’ progressive model (2 sets each time for the first 3 weeks, and 3 sets each time for the last 3 weeks) is as follows: the principle of gradual progression: to avoid over-stimulation in the early stages of training; the law of adaptation: to give the body enough time to adapt; safety considerations: to reduce the risk of exercise-related injuries. 3. Adherence: Studies have found that a SIT programme with a lower number of sets can: improve exercise adherence; reduce the dropout rate; reduce post-exercise discomfort; and enhance motivation to participate.

A six-week training intervention was conducted three times a week using a Monark 824E power bike (Sweden). To ensure the accuracy and reliability of the data, we carried out a rigorous calibration procedure for the equipment: before each experiment, the seat height, pedal resistance and electronic display were checked and calibrated to ensure that the weight of the resistance basket was accurate to ±10 g, and the accuracy of the cadence counter was calibrated; at the same time, a complete system calibration was carried out once a week, including checking the sensors and data acquisition system, and calibrating the speed and power calculation parameters. In addition, we have established a complete quality control system to record the calibration data each time and continuously monitor the stability of the equipment performance. And maintenance and recalibration will be carried out promptly in the event of any abnormalities. Research has shown that gradually increasing the amount of training is a safe and effective training strategy (Plotkin et al., 2022). Therefore, this study divided the training into an adaptation period (first 3 weeks, 2 sets each time) and an improvement period (last 3 weeks, 3 sets each time). This gradual increase in training volume ensured the safety of the participants while achieving the desired training effect. Each session involved 2-3 repetitions of 30-s Wingate sprints (Cd = 0.075, resistance on the ergometer = body mass/kg x Cd) with 4–5 m of recovery between each sprint. Prior to each test, a 5-m warm-up with unloaded cycling at 60 rpm was performed.

To ensure participant adaptation, the experiment was divided into two phases: the first 3 weeks as the adaptation phase, with 2 sets of sprints per session, and the final 3 weeks as the enhancement phase, with 3 sets of sprints per session. To monitor training adaptation, the researchers recorded the participants’ subjective feelings of fatigue (RPE scale) after each training session and the change in power output for each training set. After 3 weeks of adaptation training, all participants who completed the training showed good adaptation: the RPE scores stabilised and the power output was able to maintain at the target level, so they moved on to the improvement stage. Throughout the training process, if the participants showed signs of discomfort or overfatigue, the training intensity was adjusted accordingly or the recovery time was extended. After the experiment, post-intervention physical fitness tests were conducted to explore the effects of SIT on the physical fitness scores of male university students and to analyze the dose-response relationship of the intervention.

### 2.3 Experimental incentives

Motivation is an important factor influencing university students’ participation in physical exercise. During the experiment, the research team paid particular attention to training safety, referring to the recommendations for injury prevention during training in relevant studies (Dhahbi et al., 2024b). Intrinsic motivation significantly affects male students’ exercise behavior, but physical activity often requires extrinsic incentives (Lauderdale et al., 2015; Fletcher, 2016). Many cases show that adherence to exercise is influenced by emotional responses to exercise intensity, especially as exercise intensity increases, reducing enjoyment and pleasure (Ekkekakis et al., 2011). During the experiment, as the number of sets accumulated, participants may develop resistance or reluctance. One study showed that reducing the number of sets and duration of training can enhance positive emotions and vitality while decreasing tension, depression, and emotional instability (Nalçakan et al., 2018). The protocol used in this study reduced the number of repetitions and the duration of each session. Throughout the experiment, researchers provided verbal encouragement and rewards to maximize participant engagement and ensure the effectiveness of the intervention.

### 2.4 Recovery mechanisms

Recovery can be classified into two main categories: active recovery and passive recovery. This study referenced research on muscle adaptation to specific strength training when designing the recovery programme (Agrebi et al., 2024)to optimise recovery. However, there is no consensus on the impact of active versus passive recovery on subsequent performance. Proponents of active recovery argue that it is beneficial; for example, after a 4-m recovery between two Wingate tests, performing active recovery at 28% of maximal oxygen uptake (VO_2_max) improves performance in the s test. Active recovery may also enhance blood flow, oxygen delivery, and the resynthesis of phosphocreatine (PCr) (Spierer et al., 2004; Bogdanis et al., 1996; Dorado et al., 2004). On the other hand, advocates of passive recovery argue that, in certain situations, passive recovery has a greater positive effect than active recovery (Dupont et al., 2003; Toubekis et al., 2005). One key factor in deciding between active and passive recovery is recovery time. When two Wingate tests are conducted in close succession and recovery time is short, passive recovery may be more suitable for restoring performance (Dupont et al., 2007). Based on the experimental design of this study, with a recovery time of 4–5 m, active recovery was implemented. Participants performed 4–5 m of low-intensity exercise after each test to maximize recovery and restore body function.

### 2.5 Statistical analysis

A total of 26 participants (n = 26) were recruited for this study. However, due to injuries and discomfort experienced by some participants during the experiment, they were unable to complete the study. Therefore, 26 participants (n = 26) were included in the final data analysis. (Note: A smaller p-value and larger absolute value of the effect size (ES) indicate a greater improvement in performance due to the intervention, while a larger p-value suggests a smaller improvement.)

To control for the accumulation of type I errors due to multiple comparisons, the Holm-Bonferroni method was used to correct the p-values in this study (Holm, 1979). This method adjusts the significance level to control the overall type I error rate, which makes the interpretation of the results more rigorous.

## 3 Results

**FIGURE 1:**
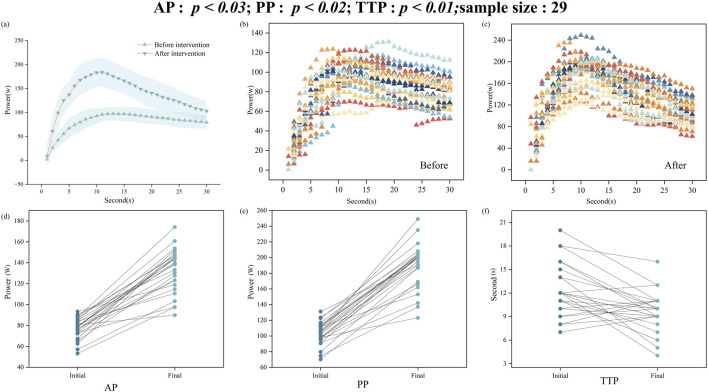
**(A)** Power output curves of all participants during the 30-s Wingate test before and after intervention. Values are presented as mean (symbols) ± SD (shaded area) for all participants (n = 26). **(B)** Individual data before intervention. **(C)** Individual data after intervention. **(D)** Changes and change amounts of Average Power (AP), **(E)** Peak Power (PP) and **(F)** Time to Peak Power (TTP) from the start to the end of the intervention.

This study significantly improved the anaerobic capacity (Table 1; Figure 1) and physical fitness test scores (Table 2) of male university students through the SIT training intervention. Table 1 and Figure 1B show a significant improvement in anaerobic capacity following the intervention. Table 1 and Figures 1D–F show a significant improvement in anaerobic capacity following the intervention. Figure 1A presents the mean power output curves with standard deviation (shaded area) during the 30-s Wingate test before and after intervention. Figures 1B, C display the individual power output curves of all 26 participants during the 30-s Wingate test before and after intervention respectively, showing the detailed performance changes of each participant. These curves clearly demonstrate an overall enhancement in power output throughout the entire test duration, with notably higher values in the post-intervention curves. To control the type I error rate in multiple comparisons, this study performed Holm-Bonferroni correction on the p-values of all test indicators (including anaerobic capacity indicators and physical fitness test indicators, a total of 9). The specific correction method is as follows: First, the p-values of the nine tests are sorted in ascending order, and then the significance level of the *k*th test is adjusted to 0.05/(10-k), where k is the position of the test in the sort (1 ≤ k ≤ 9). This step-by-step adjustment method can effectively control the overall type I error rate and has higher statistical efficiency than the traditional Bonferroni correction. The corrected p-values are shown in Tables 1, 2. After correction, the indicators of anaerobic capacity (AP, PP, TTP) remained significantly improved (p < 0.05), while the physical fitness test indicators all showed an improving trend, but the statistical significance changed, which suggests that we need to be more cautious when interpreting the effect of physical fitness improvement. Both Average Power (AP) and Peak Power (PP) were significantly increased after the intervention (AP: 77.4 ± 10.1 to 132.6 ± 21.1, p < 0.01, ES = 3.344; PP: 102.9 ± 14.5 to 189.5 ± 28.8, an increase of 84.2% p < 0.01, ES = 3.790), while Time to Peak Power (TTP) showed a significant decrease (12.3 ± 3.3 to 9.5 ± 2.6, p < 0.01, ES = −0.966). The increase in AP and PP reflects an improvement in power output after the intervention, and the decrease in TTP indicates a reduction in the time to reach peak power. These three indicators are important factors in Wingate testing that reflect improvements in anaerobic function. The changes in these indicators are positively related to the improvement of anaerobic capacity, demonstrating that the adjusted SIT protocol, with its frequency and number of sets, can still produce beneficial effects.

**TABLE 1:** The data are presented as mean ± standard deviation. The changes and change amounts of Average Power (AP), Peak Power (PP), and Time to Peak Power (TTP) from the start to the end of the intervention.

Measurement	Initial	Final	Change	95% CI	P	Adjusted P	ES
AP(w)	77.4 ± 10.1	132.6 ± 21.1	55.2 ± 20.1	[47.1, 63.3]	<0.01	<0.03	3.344_(large)_
PP(w)	102.9 ± 14.5	189.5 ± 28.8	86.5 ± 25.5	[76.3, 96.8]	<0.01	<0.02	3.790_(large)_
TTP(s)	12.3 ± 3.3	9.5 ± 2.6	−2.8 ± 3.4	[-4.2, −1.5]	<0.01	<0.01	−0.966_(large)_

**TABLE 2:** The data are presented as mean ± standard deviation. Changes in physical fitness test scores before and after the intervention. ES, Effect Size; BMI, Body Mass Index. The criteria for judging the effect size (ES) refer to Cohen’s standards (Cohen, 2013): |ES medium effect, and |ES| ≥ 0.8 is a large effect. |ES| < 0.2 is a trivial effect, 0.2 ≤ |ES| < 0.5 is a small effect, 0.5 ≤ |ES| < 0.8 is a large effect.

Measurement	Pre	Post	P	Change	95% CI	Adjusted P	ES
Height (cm)	174.4 ± 7.4	—	—	0.0 ± 0.0	[0.0, 0.0]	—	—
Body mass (kg)	70.6 ± 14.2	70.0 ± 12.4	0.852	−0.7 ± 2.5	[-1.6, 0.3]	1.000	−0.049 (trivial)
BMI(kg/m^2^)	23.2 ± 4.2	23.0 ± 3.6	0.840	−0.2 ± 0.8	[-0.5, 0.1]	1.000	−0.053 (trivial)
vital capacity (ml)	4476.7 ± 758.7	4660.7 ± 789.7	0.369	184.0 ± 324.8	[60.4, 307.6]	1.000	0.238 (small)
Sit forward in a sitting position (cm)	12.9 ± 6.8	15.8 ± 6.2	0.091	2.9 ± 2.9	[1.8, 4.0]	0.637	0.451 (small)
pull-up (rep)	5.4 ± 6.1	6.0 ± 6.4	0.722	0.6 ± 2.2	[-0.3, 1.4]	1.000	0.094 (trivial)
50-m run(s)	7.8 ± 0.8	7.5 ± 0.7	0.169	−0.3 ± 0.2	[-0.4, −0.2]	1.000	−0.366 (small)
Standing long jump (cm)	226.2 ± 21.9	234.9 ± 21.7	0.138	8.6 ± 4.4	[7.0, 10.3]	0.966	0.395 (small)
1000-m run(m)	4.6 ± 0.6	4.3 ± 0.6	0.034	−0.4 ± 0.2	[-0.4, −0.3]	0.238	−0.570 (medium)

Table 2 summarizes the improvements in various physical fitness test scores after the 6-week intervention. The effect sizes for changes in weight and BMI were small (weight: 70.6 ± 14.2 to 70.0 ± 12.4, a decrease of 0.9% p = 0.852, ES = −0.049 [trivial]; BMI: 23.2 ± 4.2 to 23.0 ± 3.6, p = 0.840, ES = −0.053 [trivial]). Sit-and-reach (12.9 ± 6.8 to 15.8 ± 6.2, an increase of 22.5% p = 0.091, ES = 0.451 [small]), 50-m run (7.8 ± 0.8 to 7.5 ± 0.7, a decrease of 3.8% p = 0.169, ES = −0.366 [small]), and standing long jump (226.2 ± 21.9 to 234.9 ± 21.7, an increase of 3.8% p = 0.138, ES = 0.395 [small]) did not reach statistical significance, but showed a small effect size improvement. The 1000-m running performance showed a statistically significant improvement with a medium effect size (4.6 ± 0.6 to 4.3 ± 0.6, a decrease of 8.7% p = 0.034, ES = −0.570 [medium]).

Finally, regarding vital capacity and pull-up performance, The data show a small effect size improvement in lung capacity (4476.7 ± 758.7 to 4660.7 ± 789.7, p = 0.369, ES = 0.238 [small]) and a small effect size improvement in pull-ups (5.4 ± 6.1 to 6.0 ± 6.4, p = 0.722, ES = 0.094 [trivial]).

## 4 Discussion

The aim of this study was to explore the effects of a 6-week SIT program on physical fitness test scores in male university students, as well as to investigate the dose-response relationship of adjustments made to the program. Our findings of significant improvements in physical fitness align with recent research: a 6-week sSIT study demonstrated significant enhancements in both anaerobic power (ES = 1.27–1.39) and aerobic fitness in athletes (Fang and Jiang, 2024), while a comprehensive meta-analysis has shown that SIT can effectively improve cardiorespiratory fitness, particularly when sprint durations are less than 30 s (Liang et al., 2024). The 6-week SIT program led to beneficial improvements in both body composition (BMI) and physical fitness (SLJ, 50-m sprint, 1000-m run, and Sit forward in a sitting position) indices in male students. Additionally, the adjustments made to the program received positive experimental feedback.

After multiple comparison correction, the indicators of anaerobic capacity (AP, PP, TTP) remained significantly improved, indicating that SIT has a definite effect on improving anaerobic capacity. However, there are still some limitations to this study that need to be explained: the small sample size and the fact that it is limited to male college students may affect the generalisability of the results; the intervention period was relatively short (6 weeks), which may not fully reflect the long-term training effect; due to the lack of a randomised controlled design, it is difficult to completely rule out the influence of other factors; at the same time, no follow-up observation was conducted after the training, so it is impossible to determine the sustainability of the training effect; in addition, the measurement of some physical fitness indicators may be affected by subjective factors, such as the degree of pain tolerance in the sit-and-reach test; finallywe were unable to monitor and control the diet and other physical activities of the participants during the experiment. These limitations should be improved in future studies. Nevertheless, in terms of effect size, indicators such as the 1000-m run (−0.570, medium) and sit-and-reach (0.451, small) still show a considerable improvement effect, which indicates that SIT may have practical significance for improving physical fitness.

After the 6-week intervention, body composition indices improved. Although there were no changes in participants’ height, there was a slight decrease in body weight. This change aligns with previous studies that have shown that intermittent training (IT) can improve health indices and promote weight loss. As a form of intermittent training, SIT can also enhance whole-body fat oxidation (Trapp et al., 2008; Boutcher, 2011; Naves et al., 2018). As a result, BMI scores decreased after the intervention, especially for participants with a lower BMI prior to the intervention, and more notably in overweight individuals with a BMI ≥25. This indicates that SIT can improve body composition, particularly in those with higher initial BMI scores.

This study found that SIT significantly improved anaerobic capacity and explosive strength, which may be related to the following physiological mechanisms, First, studies have shown that SIT training with a smaller work-rest ratio can significantly increase the energy contribution and utilization of the ATP-PCr system (La Monica et al., 2020). Second, SIT can promote adaptive changes in skeletal muscle endothelium and microvasculature, enhancing muscle contraction capacity (Cocks et al., 2013). These adaptive changes usually produce significant results after 4–6 weeks of training.

In contrast, the relatively limited improvement in lung capacity in this study is consistent with previous research. Studies have shown that even regular interval training requires at least 12 weeks of continuous training to produce significant improvements in lung function (Koubaa et al., 2019). The participants’ 1000-m run times improved substantially, which is consistent with previous research showing that SIT can influence endurance levels and improve short-distance long-distance running performance. However, the effects of SIT on long-distance running performance for both untrained individuals and well-trained athletes require further validation (Cicioni-Kolsky et al., 2013). The 1000-m run is closely related to both aerobic and anaerobic capacity. SIT training has been shown to increase peak oxygen uptake (VO_2_max), improve anaerobic performance, and enhance anaerobic capacity (Burgomaster et al., 2008; Farley et al., 2016; Burgomaster et al., 2005; Yin et al., 2023). Therefore, the SIT program in this study was effective in improving the 1000-m run performance of male university students.

Additionally, the 50-m sprint and Standing Long Jump (SLJ) performance also significantly improved. SIT has been shown to enhance exercise performance and improve anaerobic capacity (Whyte et al., 2010; Billat, 2001; Denham et al., 2015; Farley et al., 2016; MacDougall et al., 1998; Hazell et al., 2010), and anaerobic capacity is a major determinant of performance in high-intensity activities such as sprints and jumps (Arslan et al., 2022; Song and Deng, 2023). Thus, this study demonstrates that SIT positively influences the 50-m sprint and SLJ performance in male university students, consistent with previous studies on soccer players, where SIT enhanced their jumping and sprinting abilities (Wahl et al., 2014; Moran et al., 2019; Yang et al., 2024). Furthermore, lower limb strength significantly impacts SLJ performance. Previous research has indicated that the ankle, knee, and hip joints contribute significantly to SLJ performance (Robertson and Fleming, 1987), and SIT can effectively improve lower limb strength, ankle inversion strength, and anaerobic power (Chen and Kim, 2023). These factors collectively support the effectiveness of the SIT protocol used in this study.

Interestingly, this study also found an improvement in the Sit forward in a sitting position test, which can be attributed to an increase in participants’ pain tolerance, as supported by recent research showing that high-intensity interval training can significantly increase pain threshold (Gabbett and Oetter, 2024). Traditional factors influencing the Sit forward in a sitting position test include flexibility and joint mobility, but pain experienced during the test can also affect performance. Previous evidence suggests that physical performance declines when individuals experience pain (Brewer et al., 1990), while experienced contact sport athletes demonstrate higher pain tolerance and coping abilities (Thornton et al., 2021). Athletes with less pain experience may perceive pain as a threat or challenge (Thornton et al., 2017; Blascovich et al., 2004). When subjects perform the Sit forward in a sitting position test, the pain generated may also affect their athletic performance. However, engaging in high-level, high-intensity training and competition can increase pain tolerance (Ord and Gijsbers, 2003). The physiological basis for this adaptation has been linked to exercise-induced muscle damage and subsequent tissue adaptation (Clemente-Suárez et al., 2024), and research has shown that such adaptations typically occur within our 6-week intervention timeframe as part of the normal response to structured high-intensity training (Leite et al., 2023). It is plausible that the high-intensity “all-out” nature of the SIT program (Burgomaster et al., 2005) helped participants improve their pain tolerance, which may have contributed to the improvement in their Sit forward in a sitting position scores.

Regarding physical function indicators, the study found minimal impact on lung capacity, with a slight improvement. Vital capacity is closely associated with VO_2_max, oxygen saturation, and cardiovascular health. Previous studies have shown that both SIT and HIIT (High-Intensity Interval Training) can improve VO_2_max (Tjønna et al., 2013; Denham et al., 2015), increase oxygen saturation (Allemeier et al., 1994), and enhance cardiovascular health (Denham et al., 2015). Therefore, the positive effect of SIT on lung capacity (a measure of physical function) is supported.

This study adjusted the classic SIT protocol and applied a lower volume of training to male university students, a general population. The results show that this adjusted program still produced beneficial effects. Research on reducing SIT training volume has indicated that shortening the “all-out” intervals does not impact lactate threshold or aerobic capacity (Boyd et al., 2013; Yamagishi and Babraj, 2017), and reducing SIT work intervals by 50% does not diminish aerobic adaptations or the increase in lactate threshold and critical power (Zelt et al., 2014). Interestingly, no previous studies have explored whether lower-volume SIT can improve exercise tolerance and adherence. This study observed improved Sit forward in a sitting position performance, which we attribute to increased tolerance, suggesting that the modified lower-volume SIT program may enhance exercise tolerance and perseverance to some extent. However, further research is needed to investigate this further. Recent studies on low-volume high-intensity interval training (LV-HIIT) have shown that more training is not necessarily better, and LV-HIIT may offer better long-term adherence while still improving cardiovascular health and body composition (Poon et al., 2024). Given this information, future studies should focus on refining SIT protocols to develop more efficient, beneficial, and time-saving training plans. Based on the results of this study, we recommend introducing an improved version of SIT training into university physical education courses, with 2–3 sessions per week, each lasting 30s. If there is enough equipment, the duration can be increased to 1 min per student per session. Different training methods can be selected according to the venue conditions. Individualised adjustments should be made during training, starting with a lower intensity and dynamically adjusting the training plan according to the physical fitness level of the students. At the same time, on-site guidance by professionals should be ensured, necessary heart rate monitoring should be carried out, and attention should be paid to the safety control of the training environment. These recommendations are intended to help educators better apply SIT training to the practice of university physical education.

## 5 Conclusion

In conclusion, this study confirms that a 6-week SIT program (3 sessions per week, 2-3 sets of 30-s Wingate sprints per session) can effectively improve anaerobic capacity in male university students and positively influence their physical fitness test scores. Although the effect of the low-volume SIT programme on physical fitness indicators shows some potential, the lack of a control group for comparison and long-term follow-up data means that this finding still needs to be verified using a more rigorous research design. The adjusted SIT protocol still had a beneficial effect on anaerobic function, suggesting a connection to lower-volume training. Additionally, the significant improvement in the sit-and-reach score is an interesting finding. This study does have some limitations, including a lack of research on female university students and the absence of VO_2_max measurements due to limited laboratory equipment. In addition, this study did not set up a follow-up period after training to assess the sustainability of the training effect, which makes it impossible to understand the maintenance of the improvement effect (Mujika and Padilla, 2000). At the same time, the control of confounding variables such as daily physical activity levels and eating habits was also limited (Atkinson and Batterham, 2015), which may affect the interpretation of the results. Due to the above limitations, the results of this study can only provide a preliminary reference for the impact of SIT on physical fitness under specific conditions. Its practical application effect and promotion value need to be further verified. Future research needs to increase the follow-up period after training to assess the durability of the training effect (Stepto et al., 2012). At the same time, the monitoring of daily activities and living habits should be strengthened through the use of exercise diaries and wearable devices (Wright et al., 2017) to better control confounding variables. In addition, large-sample randomised controlled trials are conducted to increase the reliability of the research results. These improvements will help us to gain a more comprehensive understanding of the impact of SIT on the physical fitness of college students and develop more practical training programmes.

## Data Availability

The raw data supporting the conclusions of this article will be made available by the authors, without undue reservation.
